# Size sensors in bacteria, cell cycle control, and size control

**DOI:** 10.3389/fmicb.2015.00515

**Published:** 2015-05-29

**Authors:** Lydia Robert

**Affiliations:** ^1^UMR1319 Micalis, Institut National de la Recherche AgronomiqueJouy-en-Josas, France; ^2^UMR Micalis, AgroParisTechJouy-en-Josas, France; ^3^Laboratoire Jean Perrin (Université Pierre et Marie Curie-Centre National de la Recherche Scientifique UMR8237), Université Pierre et Marie CurieParis, France

**Keywords:** size control, bacterial cell cycle, replication initiation, incremental model, DnaA, FtsZ, MinCD

## Abstract

Bacteria proliferate by repetitive cycles of cellular growth and division. The progression into the cell cycle is admitted to be under the control of cell size. However, the molecular basis of this regulation is still unclear. Here I will discuss which mechanisms could allow coupling growth and division by sensing size and transmitting this information to the division machinery. Size sensors could act at different stages of the cell cycle. During septum formation, mechanisms controlling the formation of the Z ring, such as MinCD inhibition or Nucleoid Occlusion (NO) could participate in the size-dependence of the division process. In addition or alternatively, the coupling of growth and division may occur indirectly through the control of DNA replication initiation. The relative importance of these different size-sensing mechanisms could depend on the environmental and genetic context. The recent demonstration of an incremental strategy of size control in bacteria, suggests that DnaA-dependent control of replication initiation could be the major size control mechanism limiting cell size variation.

## 1. Introduction

All dividing cells have to coordinate different steps of the cell cycle, such as DNA replication, chromosome segregation, and cytokinesis. In eukaryotes, cell cycle control has been widely studied and the overall logic is well-understood (Murray and Hunt, 1993). The orderly progression in the eukaryotic cycle relies on a biochemical engine composed of cyclins and cyclin-dependendent kinases (CDKs). The periodic activity of cyclin-CDK complexes regulates the cell cycle transitions such as the initiation of DNA replication or the entry into mitosis (Murray and Hunt, 1993). In addition, specific control mechanisms, often called checkpoints, ensure that late events do not occur before the completion of earlier events (Hartwell and Weinert, 1989). In contrast, the logic of the bacterial cell cycle, even though intensively studied for more than half a century, remains unclear. Bacteria do not possess any cyclins or CDKs. Numerous regulatory mechanisms have been discovered and characterized in details, such as the control of DNA replication initiation (Katayama et al., 2010) or the SOS response, which inhibits division in case of DNA damage or replication arrest (Simmons et al., 2008). However, how these different mechanisms are organized and connected to ensure an orderly progression into the cycle remains unclear (Haeusser and Levin, 2008).

In many organisms, progression through the cycle is coupled with cellular growth. In the budding yeast *Saccharomyces cerevisiae*, the duration of the G1 phase depends on the cell size at birth, smaller cells spending a longer time in G1 (Johnston et al., 1977; Di Talia et al., 2007; Turner et al., 2012). Likewise, in the fission yeast *Schizosaccharomyces pombe*, mitotic entry is delayed in smaller cells (Fantes, 1977; Sveiczer et al., 1996). In bacteria, cell cycle progression was as well-assumed to be under size control. In 1968, building on the seminal physiological studies of Schaechter et al. (1958) and Cooper and Helmstetter (1968) on *Salmonella typhimurium* and *Escherichia coli*, Donachie showed that at the population level, the average initiation mass, i.e., the ratio of cell mass to the number of replication origins is constant regardless of the growth rate and culture medium (Donachie, 1968). This lead him to propose a size control mechanism at the single cell level, in which the cell initiates DNA replication when it reaches a critical size, and divides a constant time after initiation. Although widely accepted for decades, this model became controversial in recent years (Voorn et al., 1993; Wold et al., 1994; Boye et al., 1996; Bates and Kleckner, 2005; Chien et al., 2012; Hill et al., 2012). Nevertheless, definitive evidence for size control in bacteria was provided by recent studies using quantitative data on single cell growth and division in both *E. coli, Bacillus subtilis*, and *Caulobacter crescentus* (Campos et al., 2014; Osella et al., 2014; Robert et al., 2014; Soifer et al., 2014; Taheri-Araghi et al., 2015).

However, no size-sensing module has been identified so far, and the molecular basis of size control stays unclear. Cells could sense their length, volume or mass. Here I use cell size as a catch-all descriptor and describe how cell size could be sensed through different pathways involved in the control of cytokinesis, chromosome segregation or replication initiation. Importantly, the potential size-sensing mechanisms are not mutually exclusive and size control could be implemented redundantly at several cell cycle stages. Such redundancy has been evidenced in the fission yeast *S. pombe*. In addition to the major size control acting at mitosis entry (Fantes and Nurse, 1977), a second size control mechanism acts at the G1/S transition (Fantes and Nurse, 1978). This second size-control is usually invisible since the G2/M control produces cells whose size at birth already exceeds the requirement of the G1/S control. Nevertheless, this second mechanism can be revealed when the primary G2/M control is perturbed, such as in the *wee1* mutant (Fantes and Nurse, 1978). *S. cerevisiae* has also been proposed to exhibit an usually invisible size-control, acting at the G2/M transition (Murray and Hunt, 1993; Turner et al., 2012). Several size checkpoints could as well-exist in bacteria, where the cytokinesis, the chromosome segregation and the initiation of replication might all depend on cell size. As in yeast, the most stringent of these size control mechanisms would be responsible for the limitation of the variations of the cell size in bacteria. Interestingly, recent experimental results on size control in bacteria argue against the current critical size paradigm and suggest an incremental strategy: division does not occur at a critical size but rather when a constant size has been added to the size at birth (Campos et al., 2014; Soifer et al., 2014; Taheri-Araghi et al., 2015). Such phenomenological description sheds a new light on the mechanism limiting cell size variations. Therefore, after a description of all the potential size-sensing mechanisms involved in the control of cytokinesis, chromosome segregation and replication initiation, I will discuss which of these mechanisms could be responsible for the limitation of cell size variations, in light of this incremental principle.

## 2. The min system: a geometric size-sensor controlling cytokinesis?

A critical event in the bacterial division process is the polymerization of the tubulin-like protein FtsZ into an annular structure called the Z ring, which locates the division site and recruits the numerous proteins required to carry out cytokinesis (Bi and Lutkenhaus, 1991; Addinall and Holland, 2002; Margolin, 2005; Harry et al., 2006). In *E. coli* and *B. subtilis*, the positioning of the Z ring has been widely studied. It is generally assumed to rely on two inhibitory systems: Nucleoid Occlusion (NO) and the Min system (Lutkenhaus, 2007; Wu and Errington, 2011). The latter is based on the protein complex MinCD, an inhibitor of FtsZ polymerization that concentrates at the cell ends, and is therefore essential to prevent polar divisions (Adler et al., 1967; De Boer et al., 1989; Lutkenhaus, 2007). In *E. coli*, the localization pattern of MinCD emerges from remarkable oscillations of the complex from pole to pole, which create over time a gradient of concentration showing a minimum around mid-cell (Hu and Lutkenhaus, 1999; Raskin and de Boer, 1999; Hale et al., 2001). In contrast, in *B. subtilis* MinCD does not oscillate and the gradient is static (Lutkenhaus, 2007). In both organisms, the concentration of the MinCD complex along the cell axis may vary with cell length and MinCD has therefore been proposed to serve as a size ruler (Raskin and de Boer, 1999). Nevertheless, there is no experimental evidence yet allowing confirming or invalidating this hypothesis.

The function and localization of MinCD is very similar to those of the Pom1 kinase in the fission yeast *S.pombe* (Padte et al., 2006; Huang et al., 2007). This kinase exhibits a gradient of concentration in the cell, with maxima at cell ends and a minimum around mid-cell. This localization allows Pom1 to prevent the assembly of the division septum at the cell ends. It was also recently suggested to serve as a size sensing device responsible for size control at mitotic entry. The work of two different teams published in 2009 established that Pom1 is a dose-dependent inhibitor of mitosis, whose concentration at the cell middle decreases when the cells elongate (Martin and Berthelot-Grosjean, 2009; Moseley et al., 2009). Pom1 was shown to regulate the G2/M transition by inhibiting mitotic activators localized at the middle of the cells. The following model of size-dependent G2/M transition was proposed: when the cells are short, the high concentration of Pom1 at mid-cell where it can interact with mitotic regulators inhibits mitotic entry. When the cell grows, this inhibition progressively weakens as the concentration of Pom1 at mid-cell decreases. Pom1 was therefore proposed to be a dedicated size sensor involved in cell cycle control. This discovery appeared promising for understanding the molecular basis of size control. Nevertheless, a few years later, one of the team involved in this discovery presented evidence that Pom1 is in fact not the size sensor but rather a modulator that affects the link between the measured size and the division probability, thus changing the size threshold at mitosis (Novák, 2013; Wood and Nurse, 2013). This conclusion was supported by the behavior of the *pom1*Δmutant, which is shorter but exhibits a wild type correction for cell size fluctuations, compressing or extending the duration of the cycle according to the cell's size at birth (Wood and Nurse, 2013).

Bacteria possess some geometric sensors similar to Pom1. In *E. coli* and *B. subtilis*, the potential Pom1-like geometric sensor is the MinCD complex. In *C. crescentus*, which has no MinCD orthologs, a gradient of the inhibitor of FtsZ polymerization MipZ emanates from the cell poles after the segregation of the replication origins (Goley et al., 2007). The mechanism that was proposed for size-sensing through Pom1 could in principle apply to its bacterial counterparts: cell elongation could lead to a decrease of MinCD/MipZ concentration in the cell middle, thus triggering the formation of the Z ring. Alternatively, these regulators could be, like Pom1, modulators of cell size at division.

## 3. FtsZ, a size sensor?

In the fission yeast, very recent results suggest that the cdr2 mitotic activator regulated by Pom1 could be the size sensor controlling mitotic entry (Pan et al., 2014). Cdr2 is localized in a band of cortical nodes at mid-cell, where it accumulates when the cell elongates. Pan et al. therefore proposed that a critical cdr2 concentration is attained when the cell reaches a critical size and triggers mitotic entry. The bacterial FtsZ protein, the target of MinCD regulation, has also been proposed to act as a size sensor (Chien et al., 2012). In this model, division would occur when the amount of FtsZ inside the cell reaches a threshold. In support of this hypothesis, FtsZ levels are known to modulate the size at division in *E. coli* (Chien et al., 2012). In *C. crescentus*, FtsZ expression and degradation are regulated during the cell cycle. As a consequence, the concentration oscillates during the cycle with a maximum at the onset of constriction, after which FtsZ is degraded (Quardokus et al., 1996; Kelly et al., 1998). This dynamics is in agreement with division being triggered by a critical level of FtsZ. In contrast, in *E. coli* and *B. subtilis*, FtsZ is constitutively expressed and its concentration is constant throughout the cycle. Its total amount is therefore proportional to cell size.

The capacity of the yeast protein Cdr2 to serve as a size-sensor mainly relies on its localization, so that the local concentration at mid-cell increases when cells elongate. Similarly, specific localization of FtsZ could be responsible for a size-dependent local concentration at mid-cell. In addition to ring structures, FtsZ can assemble into helices in *E. coli* and *B. subtilis* (Ben-Yehuda and Losick, 2002; Thanedar and Margolin, 2004; Harry et al., 2006). FtsZ has also been shown to oscillate from one pole to the other, out of phase with the Min system (Thanedar and Margolin, 2004; Bisicchia et al., 2013). The Z ring may therefore form from the reorganization of moving helices. However, helical structures of FtsZ have still to be confirmed. In particular, such helical structures can be artifacts of fluorescent fusions, as demonstrated for the MreB protein (Domínguez Escobar et al., 2011; Van Teeffelen et al., 2011; Swulius and Jensen, 2012). Therefore, the localization of FtsZ is still unclear, but some size-dependence of the local concentration at mid-cell is possible, leading to a critical size for Z ring formation. Importantly, in *E. coli* and *B. subtilis* the ring is known to form well before constriction (Den Blaauwen et al., 1999). In addition, recent data suggests that at the single cell level, the timing of its formation is independent of the timing of constriction and the cell length at birth (Tsukanov et al., 2011). It is therefore possible that once the ring is formed, the amount of FtsZ it contains increases up to a threshold where the ring becomes able to recruit all the downstream components of the divisome and constrict. In this case, division timing could depend on cell size through FtsZ accumulation inside the ring.

## 4. Chromosome segregation: a size-dependent event?

Chromosome segregation occurs in three steps: separation of the newly replicated origins, bulk chromosome segregation and separation of the replicated termini (Wang et al., 2013). Bulk chromosome segregation appears as an important transition, leading to the appearance of a DNA free space at mid-cell and relief of the division inhibition mediated by Nucleoid Occlusion (NO). NO prevents the formation of the Z ring in the vicinity of the chromosome (Mulder and Woldringh, 1989; Wu and Errington, 2011). The mechanism of NO is not fully elucidated yet but some molecular bases have been provided by the discovery of the Noc protein of *B. subtilis* and SlmA protein of *E. coli*, which associate with DNA and inhibit FtsZ polymerization (Wu and Errington, 2004; Bernhardt and De Boer, 2005). Interestingly, these NO proteins bind to specific DNA sequences that decorate a large portion of the chromosome but are absent from the Ter region (Wu and Errington, 2011). Therefore, NO proteins are largely removed from mid-cell following bulk chromosome segregation.

The mechanism underlying bulk chromosome segregation are still unclear but entropic forces have been proposed to either drive or facilitate segregation (Jun and Wright, 2010; Di Ventura et al., 2013). Such forces can in theory segregate mixed chains of polymers in a situation of confinement. A single *E. coli* chromosome is more than 1 mm in length and has to be compacted more than 1000-fold to fit inside the cell (Wang et al., 2013). Replicated chromosomes are thus confined inside the cell, whose shape and size will determine the intensity of the entropic forces. Importantly, if segregation is entropy-driven, it should be easier in longer cells. During cell elongation, the intensity of the entropic forces increases, which could lead to size-dependent segregation. In agreement with this hypothesis, experimental results on cell cycle progression in single cells suggest that the timing of nucleoid splitting depends on cell growth rather than on replication progression (Bates and Kleckner, 2005). Therefore, if driven by entropic forces, the very act of segregation could be a size-dependent signal, transmitted to the division machinery through the relief of NO.

Interestingly, the MinCD gradient has recently been shown to be involved in chromosome segregation (Di Ventura et al., 2013). MinD was shown to bind DNA and tether it to the cell membrane. The Min system therefore provides a gradient of membrane sites for DNA attachment. Computer simulations showed that entropic forces alone could not ensure full chromosome segregation. In contrast, full segregation would be possible if chromosomes repeatedly bind to membrane-associated sites forming a gradient emanating from the pole, such as provided by MinD. The concentration of MinD along the cell axis is likely to depend on cell length, as was demonstrated for Pom1 in the fission yeast (Martin and Berthelot-Grosjean, 2009; Moseley et al., 2009). Its involvement in segregation suggests another potential size-dependence for chromosome segregation.

## 5. The initiation of replication: size-sensing through DnaA?

In the long-standing model of bacterial cell cycle initially proposed by Donachie, the coordination between growth, replication and division is mainly achieved through the control of replication initiation : the cell grows until reaching a critical size at which point replication is initiated and division occurs at a fixed time after initiation (Donachie, 1968). Donachie proposed that replication initiation could be controlled by a positive regulator, which accumulates proportionally to cell size and triggers initiation when reaching a critical amount. This critical amount would therefore correspond to a critical size. Later, DnaA was identified as the initiator protein in bacteria and has therefore been the natural candidate for Donachie's positive regulator (Løbner-Olesen et al., 1989).

I will focus here on *E. coli*, where replication initiation has been most studied and best characterized. In *E. coli*, the initiator DnaA is active when bound with ATP and inactive when bound with ADP. Active DnaA binds to specific DNA sequences in the replication origin region, leading to unwinding of the DNA sister strands in a neighboring AT-rich region and loading of the replisome (Katayama et al., 2010). This initiation event is tightly controlled in order to occur once and only once in each cell cycle. In particular, several mechanisms ensure the inactivation of DnaA following initiation, in order to prevent immediate reinitiation. These mechanisms include seqA-dependent sequestration of OriC and transcriptional repression of the *dnaA* gene (Campbell and Kleckner, 1990), titration of DnaA to the datA chromosomal locus (Kitagawa et al., 1996), and DnaA inactivation through RIDA, a replication-coupled mechanism involving a complex of Hda, ADP, and the DNA polymerase sliding clamp (Kato and Katayama, 2001; Katayama et al., 2010). All these mechanisms ensure that replication initiation occurs only once in the cell cycle. After this wave of inactivation, the newly produced DnaA, which is rapidly converted to ATP-DnaA (Messer, 2002), accumulates in the cell until the next initiation event. In addition, some reactivation mechanisms convert ADP-DnaA to ATP-DnaA (Katayama et al., 2010). A mechanism involving cardiolipin, a membrane phospholipid, has been suggested (Sekimizu and Kornberg, 1988), and two specific DNA sequence, DARS1 and DARS2, have been demonstrated to promote the conversion of ADP-DnaA to ATP-DnaA (Fujimitsu et al., 2009).

As an outcome, the cellular level of ATP-DnaA oscillates during the cell cycle, with a maximum at the time of initiation (Kurokawa et al., 1999). Initiation has therefore been proposed to occur when the amount of ATP-DnaA reaches a threshold. Interestingly, when DnaA is overexpressed several folds, the initiation timing is only slightly perturbed (Atlung et al., 1987; Kurokawa et al., 1999). In these conditions, it was shown that the proportion of the ATP and ADP-bound forms is unchanged (Kurokawa et al., 1999). Since these two forms might compete for binding DNA, initiation could be triggered when the ratio of ATP-DnaA to ADP-DnaA reaches a threshold (Donachie and Blakely, 2003). Thus, a simple model of initiation control, developed in Donachie and Blakely (2003), postulates that DnaA is synthesized constitutively, so that its total amount is proportional to cell mass, and immediately binds ATP (Messer, 2002). The ratio of ATP to ADP bound DnaA therefore would increase in parallel with cell size, until it reaches a threshold and triggers initiation of replication, leading to the inactivation of DnaA. In this model, the constitutive expression of DnaA and its immediate binding to ATP leads to an amount of active DnaA and a ratio of the active to inactive form both increasing linearly with cell size after replication initiation. It is still unclear how this linear relation could be affected by the reactivation of DnaA during the cycle, which is not taken into account in this simple model.

## 6. Recent revision of the critical size paradigm of size control: the incremental model

In both yeast and bacteria, substantial correlations are observed at the single cell level between size at birth and size at division (Campos et al., 2014; Soifer et al., 2014; Taheri-Araghi et al., 2015). Such correlations argue against the classical model of cell size control in which division occurs at a critical size, up to some noise, with no memory of the size at birth. Indeed both yeasts and bacteria show some memory between one generation and the next: the cell's size at division is correlated to its size at birth, i.e., to the size at division of its mother.

Such a memory could in principle be the result of epigenetic inheritance of some molecules involved in the determination of the critical size. Gene expression naturally undergoes random fluctuations whose time scale is usually larger than a generation time. Such slow fluctuations can therefore generate correlations between the abundance of a protein in a cell and in its daughter (Rosenfeld et al., 2005; Longo and Hasty, 2006). When considering a molecular pathway involved in size measurement and control of cell cycle progression, such simple epigenetic memory would be likely to introduce some correlations throughout generations, such as between the size at division of a cell and its daughter's. The simple critical size model can be modified to take such memory into account. For instance an additional structuring variable can be added in the equations of the so-called sloppy size control model, using a division rate depending not only on the cell size but also on its size at birth (Osella et al., 2014). Using another mathematical framework, size at division can be described using an autoregressive model (Amir, 2014).

Strikingly, in very different organisms such as *E. coli, B. subtilis, C. crescentus*, and *S. cerevisiae*, the dependence of size at division on size at birth is the same, a linear relationship with slope one (Campos et al., 2014; Soifer et al., 2014; Taheri-Araghi et al., 2015). It is worth noting that the case of *C. crescentus* is currently debated, since another dependence have been suggested by Iyer-Biswas et al., who proposed that the size at division is a multiple (≈1.8) of the size at birth, indicating a timer mechanism of division control (Iyer-Biswas et al., 2014). Nevertheless, the data obtained by Iyer-Biswas et al. was then analyzed independently by Jun et al. in a way similar to the analysis performed in *E. coli, B. subtilis*, and *S. cerevisiae*, giving a linear relationship with slope ≈1.2 (Jun and Taheri-Araghi, 2015). The linear relationship with slope one is precisely the prediction of the so-called incremental model, where a cell tries to add a constant volume between birth and division (Sompayrac and Maaløe, 1973; Voorn et al., 1993; Amir, 2014). Therefore, even though some memory can be caused by slow fluctuations in gene expression, the value of the memory exhibited by the size at division through the successive generations and its conservation among several very divergent organisms suggest a more profound revision of the classical critical size model: the cell does not divide at a critical size but tries to add a constant size to its size at birth.

## 7. Candidate sensors for an incremental measure of cell size and possible mechanisms limiting cell size variations

### 7.1. Control of cytokinesis and chromosome segregation

Although the interpretation of the experimental data in terms of the incremental model cannot conclude on the underlying molecular mechanisms, it offers a phenomenological description and sheds a new light on the possible mechanisms limiting cell size variations. As detailed in the previous sections, several mechanisms may be responsible for the size-sensing and the size-dependent control of cytokinesis, chromosome segregation and DNA replication initiation. Among these mechanisms, one may lead to the limitation of cell size variations. This mechanism should implement an incremental strategy.

Potential geometric sensors such as MinCD are unlikely to implement an incremental strategy: they could link the instantaneous division probability with the current cell size but could hardly measure a size increment since birth. Importantly, Campos et al. showed that a Δ*minC* mutant of *E. coli* also exhibits an incremental strategy of size control, therefore demonstrating that the Min system does not play a crucial role in sensing the size increment (Campos et al., 2014).

Likewise, the process of chromosome segregation may be size-dependent but is unlikely to be related to a size increment. Entropic forces potentially driving or facilitating segregation could depend on the instantaneous cell size and geometry but hardly on the difference between the instantaneous size and the size at a previous cell cycle event. Likewise, the potential involvement of MinCD in segregation would rather create a dependence on the instantaneous cell size than on a size increment.

In *E. coli* and *B. subtilis*, the concentration of FtsZ is constant during the cell cycle. Its total amount is therefore proportional to cell size and not to the size increment since birth. FtsZ is thus unlikely to be a size increment sensor in these organisms. The situation is different for *C. crescentus*, where FtsZ is degraded at the onset of constriction (Quardokus et al., 1996; Kelly et al., 1998). The FtsZ-dependent size measure would therefore be reset at the end of the cycle when FtsZ is degraded. FtsZ levels could therefore in principle be correlated to the increment of size since birth in this organism.

Therefore, in *E. coli* and *B. subtilis* the limitation of cell size variations seems unlikely to result from size control at the level of cytokinesis or chromosome segregation. In contrast, in *C. crescentus* FtsZ could perform a measure of size increment.

### 7.2. Control of replication initiation

The incremental model postulates a constant size increment between two successive events of the cell cycle, such as between birth and division or between an initiation event and the next one. Interestingly, Campos et al. studied the possibility that the size increment could be applied at a cell cycle event other than division, such as replication initiation (Campos et al., 2014). They performed simulations of such a “phase-shifted” model and found that it was not compatible with their data on *E. coli* and *C. crescentus*. In particular, their model produced a negative correlation between the size increment (between birth and division) and the size at birth, whereas no such correlation exists in the data. Nevertheless, recent work by Ho and Amir (in this Frontiers Research Topic: The Bacterial Cell: Coupling between Growth, Nucleoid Replication, Cell Division and Shape) shows that this correlation strongly depends on the variability in the durations from replication initiation to division. If this variability is small compared to the variability in the duration of the whole cell cycle (less than 30 percent), then the correlation is close to zero, as experimentally observed. Campos et al. also report that their phase-shifted model produces abnormal cell size distributions. Nevertheless, using a different phase-shifted model, Ho and Amir show that cell size can be robustly regulated. Therefore, the results of the simulations of the “phase shifted” model of Campos et al. cannot be used to rule out the possibility of size control at the level of replication initiation.

**Figure 1:**
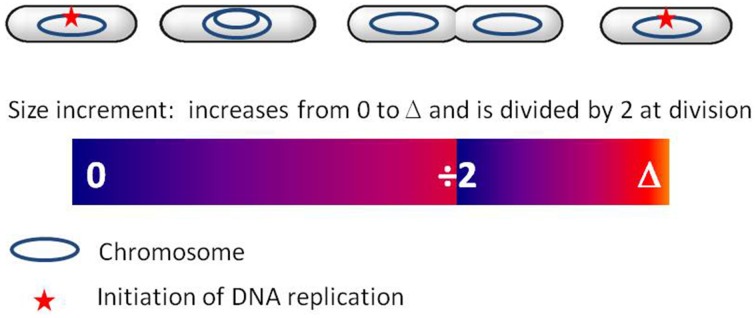
**Incremental model for cell size control at replication initiation in bacteria (see also Amir, 2014)**. For simplicity, a slowly growing *E. coli* cell is represented. The colored bar represents the dynamics of the size increment S through the cell cycle. After initiation of replication, S increases until division. At division, *S* is shared between the two daughter cells. In each daughter cell, S then increases up to the critical value Δ, leading to replication initiation and resetting of the size increment (*S*=0).

When *E. coli* cells are shifted from a poor medium to a rich medium, the rate of mass increase immediately changes whereas the rate of cell division is maintained at the pre-shift value for a lag of approximately 60 min, corresponding to the constant C + D period (Kjeldgaard et al., 1958; Cooper, 1969). This phenomenon, called rate maintenance supports the hypothesis of size control acting at the level of replication initiation (Cooper, 1969), such as proposed in Donachie's model (Donachie, 1968; Donachie and Blakely, 2003). Also in support of this hypothesis, cell size is exponentially dependent on growth rate (Schaechter et al., 1958), with an exponent of 60 min (i.e., C + D period). This relation can be easily derived in the framework of the incremental model when the size increment is added between two successive replication initiation events, as shown in Amir (2014). If initiation is triggered by the size-dependent amount of active DnaA, its subsequent inactivation would reset the size measure at each initiation event. Donachie's model could therefore be revisited to account for the incremental strategy: initiation would be triggered not at a critical size but when a critical size has been added since the last initiation event (see Figure 1). Division would follow a constant time after initiation, through an as-yet unknown mechanism. As mentioned above, in addition to the ATP-DnaA formed by *de novo* DnaA expression, ADP-DnaA can be partly reactivated during the cycle. How the amount of ATP-DnaA varies with cell mass between two initiation events is therefore not completely clear. In addition, initiation may be triggered by other DnaA-dependent variables, such as the ratio of the active to inactive forms (Donachie and Blakely, 2003). It is still unclear what is the triggering signal for initiation and how it is linked to cell size. Nevertheless, the inactivation of DnaA following initiation is crucial in initiation control and appears as an interesting basis for the implementation of an incremental size measure. The incremental model can satisfyingly describe *E. coli, B. subtilis*, and *C. crescentus* as well as the budding yeast *S. cerevisiae*. The conservation of this size control strategy among widely divergent organisms is striking and suggests some common organizing principle. Interestingly, even though the molecular mechanisms involved in replication initiation are different among these organisms, common control principles can be found. For instance the negative regulation of OriC after initiation is common to *E. coli, B. subtilis*, and *C. crescentus* (Katayama et al., 2010). Also, the principle of RIDA, which inactivates DnaA in *E. coli* in a replication-coupled manner through the action of the polymerase sliding clamp, is widely conserved (Katayama et al., 2010). The clamp-mediated inactivation of initiation proteins has also been demonstrated in *B. subtilis* (Soufo et al., 2008), *C. crescentus* (Collier and Shapiro, 2009), and in several eukaryotic organisms (Arias and Walter, 2006; Nishitani et al., 2006; Katayama et al., 2010). For all these organisms the initiation potential may therefore fluctuate in the cell in a way similar to the DnaA-related signal in *E. coli*. This may lead to a common size control strategy, such as described by the incremental model.

## 8. Conclusion

The identification of a size-sensing molecule has been a long lasting quest and has proven surprisingly difficult, as evidenced by the recent controversy on the role of Pom1 in the fission yeast. This might indicate that size-sensing is generally not the function of a single molecule but is rather a systems-level property. In other words, several proteins inside a regulatory pathway may participate to the size-dependence of a cell cycle event. As an example, in *E. coli* the local concentrations of MinCD and FtsZ at mid-cell might both change when the cell elongates and could in concert determine the size-dependence of cytokinesis. Importantly, numerous cell cycle regulators exhibit specific localization patterns, such as CtrA and MipZ in *C. crescentus*, MinCD, SlmA, DnaA in *E. coli* (Bernhardt and De Boer, 2005; Goley et al., 2007; Lutkenhaus, 2007; Boeneman et al., 2009; Nozaki et al., 2009). Such subcellular localization can readily result in size-dependent local concentration. When a protein is constitutively expressed, its total amount is proportional to cell size. If such a protein is localized in a subcellular volume that does not change proportionally to the total volume when the cell grows, its local concentration is size-dependent. As a simple example, if the subcellular volume is constant, the protein concentration is proportional to the total cell volume. Several regulators in a single pathway can be localized, in particular since some events such as cytokinesis are spatially controlled, and several size-dependent signals may therefore co-exist, leading to a complex size control of the event.

Here I described several regulatory pathways that could lead to size-dependent regulation of replication initiation, chromosome segregation and cytokinesis. In principle they could all be responsible for cell size control, i.e., for the observed dependence of the interdivision time on the size at birth at the single cell level. Old physiology experiments, in particular demonstrating the “rate maintenance” phenomenon Kjeldgaard et al., 1958; Cooper, 1969), suggest that size control in *E. coli* acts primarily at the level of replication initiation (Cooper, 1969). In agreement with this hypothesis, the dynamics of DnaA activity could potentially explain the observed incremental strategy for size control. The inactivation of DnaA following initiation could reset some DnaA-dependent signal which could therefore be linked to a size increment. In addition, the widely conserved principles underlying replication initiation control are in agreement with the common incremental strategy found in three divergent bacteria, as well as in a unicellular eukaryotic organism. In the budding yeast, size control is known to act primarily at the Start transition, i.e., the onset of replication, with a modulation of the G1 duration according to cell size at birth (Johnston et al., 1977; Turner et al., 2012). Experimental data are also compatible with size control acting slightly earlier, for instance at the level of origin licensing (Soifer et al., 2014). In contrast, in *S. pombe*, size control is known to act primarily at the G2/M transition (Fantes and Nurse, 1977; Turner et al., 2012). Interestingly, a recent analysis of single cell growth and division in this organism indicate that it does not follow the incremental model (Nobs and Maerkl, 2014), suggesting that the incremental strategy could be a property of the size control provided by the regulatory mechanisms of replication initiation. It would be interesting to study at the single cell level the *wee1* mutant of *S. pombe*, which exhibits size control at the G1/S transition and determine whether size control follows an incremental strategy. In bacteria, DnaA-dependent initiation may provide the principal mechanism limiting cell size variations. Other cell cycle transitions may be size dependent, such as cytokinesis or chromosome segregation, leading to secondary size control mechanisms that could be revealed in specific environmental or genetic contexts, as demonstrated in yeasts.

### Conflict of interest statement

The author declares that the research was conducted in the absence of any commercial or financial relationships that could be construed as a potential conflict of interest.
